# Reduction of organelle motility by removal of potassium and other solutes

**DOI:** 10.1371/journal.pone.0184898

**Published:** 2017-09-18

**Authors:** John W. Murray, David Yin, Allan W. Wolkoff

**Affiliations:** 1 Marion Bessin Liver Research Center, Division of Gastroenterology and Liver Diseases, Albert Einstein College of Medicine and Montefiore Medical Center, Bronx, New York, United States of America; 2 Department of Anatomy and Structural Biology, Albert Einstein College of Medicine and Montefiore Medical Center, Bronx, New York, United States of America; Institut de Genetique et Developpement de Rennes, FRANCE

## Abstract

There are surprisingly few studies that describe how the composition of cell culture medium may affect the trafficking of organelles. Here we utilize time lapse multi-channel fluorescent imaging to show that short term exposure of Huh-7 cells to medium lacking potassium, sodium, or chloride strongly reduces but does not eliminate the characteristic back and forth and cell-traversing movement of fluorescent EGF (FL-EGF) containing organelles. We focused on potassium because of its relatively low abundance in media and serum and its energy requiring accumulation into cells. Upon exposure to potassium free medium, organelle motility declined steadily through 90 min and then persisted at a low level. Reduced motility was confirmed in 5 independent cell lines and for organelles of the endocytic pathway (FL-EGF and Lysotracker), autophagosomes (LC3-GFP), and mitochondria (TMRE). As has been previously established, potassium free medium also inhibited endocytosis. We expected that diminished cellular metabolism would precede loss of organelle motility. However, extracellular flux analysis showed near normal mitochondrial oxygen consumption and only a small decrease in extracellular acidification, the latter suggesting decreased glycolysis or proton efflux. Other energy dependent activities such as the accumulation of Lysotracker, TMRE, DiBAC4(3), and the exclusion of propidium iodide remained intact, as did the microtubule cytoskeleton. We took advantage of cell free in vitro motility assays and found that removal of potassium or sodium from the reconstituted cytosolic medium decreased the movement of endosomes on purified microtubules. The results indicate that although changes in proton homeostasis and cell energetics under solute depletion are not fully understood, potassium as well as sodium appear to be directly required by the motile machinery of organelles for optimal trafficking.

## Introduction

Cytoskeletal based movement of organelles is associated with many fundamental activities such as the delivery of biosynthetic cargo to the plasma membrane, the sorting of endocytic content to lysosomes, and maintenance of mitochondrial health and distribution through fusion and fission [1–3]. The importance of organelle movement is heightened in neuronal tissues where for example, mitochondria may travel more than a meter to reach peripheral destinations [4, 5]. Organelle traffic can be visualized and quantified by many techniques. For instance, exposure of cells to fluorescent ligand or to lysosomotropic dyes reveals a galaxy-like array of endosome and lysosome puncta and tubules undergoing back and forth bursts of movement with speeds of up to 2.5 μm/sec and continuous runs of more than 10 μm [6, 7]. To visualize and quantify the movement of intracellular organelles as well as endocytosis itself, we utilized a fluorescent version of well known endocytic ligand, EGF. Epidermal growth factor (EGF) is a peptide growth factor that binds to the cell surface EGF receptor (EGFR) and induces receptor dimerization and activation of the EGFR cytosolic tyrosine kinase. This stimulates a host of cellular activities including DNA synthesis, cell proliferation, and migration. At the same time, receptor binding stimulates endocytosis of EGF-EGFR, resulting in the formation of cytosolic vesicles and tubules containing EGF-EGFR within their lumen. These undergo endocytic processing and maturation through early endosomes, the recycling compartment, and culminating in lysosomes where EGF-EGFR is degraded and its cellular signaling is quenched [8]. When EGFR-expressing cells are exposed to FL-EGF, the majority of the fluorescence will first be visible at the plasma membrane (~0–3 min) and then within internalized endocytic and recycling endosomes and finally within lysosomes. Beyond 30 min of endocytic processing, internalized EGF is typically found in lysosomes [9], but this may be affected by the experimental manipulations presented below, and images and data containing FL-EGF may represent lysosomes as well as other organelles along this pathway.

Movement of organelles can be reduced or eliminated by depolymerization of microtubules, depletion of ATP [7, 10, 11], reduction in temperature [12, 13], addition of inhibitors or antibodies to motor proteins [14, 15], genetic mutation of motors, or overexpression of motor protein cofactors, such as dynamitin [15, 16]. In addition to these experimental maneuvers, we considered whether the composition of extracellular medium could have an effect. Specifically we wondered whether low levels of extracellular K^+^ or other solutes could affect organelle movement since depletion of K^+^ had previously been shown to inhibit endocytosis and cell polarization [17, 18]. Energy dependent accumulation of K^+^ is an integral component of cellular function. Mammalian cells concentrate K^+^ almost 40 fold, from approximately 4 mM present in serum to 150 mM found in cytosol. This stems from the energy dependent asymmetric exchange of Na^+^ and K^+^ by the Na^+^/K^+^ ATPase, which establishes the plasma membrane electric potential [19, 20]. Although K^+^ is essential for growth of cells in culture [21], short term reduction in K^+^ is non-lethal, and its primary metabolic effect is inhibition of protein synthesis [19]. Larkin et al [17] had also found that K^+^ depletion specifically blocks receptor mediated endocytosis and the associated focal accumulation of clathrin at the plasma membrane without blocking the binding of ligand to its receptor. Yet these studies did not indicate whether intracellular vesicle trafficking is affected. In this report we explored whether the removal of extracellular K^+^ and other solutes may affect organelle traffic, how cellular metabolism may be altered, and whether changes would also occur within cell free motility assays.

## Materials and methods

### Cell culture and chemicals

Reagents were from Sigma-Aldrich (St. Louis, MO) unless noted. 8-well coverslip-bottomed chambers were from Nunc Lab-Tek (Thermofisher #15541), and 4-well chambers from In Vitro Scientific (Sunnyvale, CA). All animal procedures were approved by the University Animal Use Committee of Albert Einstein College of Medicine. Cells were cultured on DMEM (Thermofisher #31053028) supplemented with 10% FBS, penicillin / streptomycin, and Glutamax. Cell culture and all incubations in DMEM were in 5% CO_2_. Incubations in non bicarbonate containing medium were in the absence of CO_2_. Huh-7, MDCK cells were authenticated and provided by the Animal Models, Stem Cells, and Therapy Core at the Albert Einstein College of Medicine, 3T3 cells stably transfected with mCherry-GFP-LC3 were a gift from the laboratory of Ana Maria Cuervo (Albert Einstein College of Medicine) [22]. HeLa cells were from the Wolkoff lab repository [23], 293FT cells were authenticated and provided by the Gene Therapy Core at the Albert Einstein College of Medicine.

### Live cell organelle motility protocol

The cells were plated at 50–70% confluence in DMEM and the following day exposed to Alexa 647 labeled EGF (Thermo Fisher # E35351) for 10 min followed by 10 μM Hoechst nuclear stain for 5 min followed by washing 5X in live cell medium (120.8 mM NaCl, 2.5 mM KCl, 1 mM MgCl_2_, 1.8 mM CaCL_2_, 25 mM Glucose, 20 mM Hepes-Tris (12.5 mM Hepes, 7.5 mM Tris), pH 7.4) or solute substituted medium. Care was used to avoid cell drying or contact with air interface, which can damage cells. Note that Hepes and Tris were titrated to set the pH, addition of KOH, NaOH, or HCl was avoided. Cells were then incubated 15–180 min at 37°C, and then imaged at 1 frame / 2 sec for 1 min, generating 31 frames of moving organelle data. For motility of other organelles, the above protocol was followed but FL-EGF was avoided, cells were incubated 90 min in medium, and either Lysotracker Red DN99 (70 nM, Thermo Fisher L752) or TMRE (5 nM or 30 nM, Anaspec # AS-88061) was added and incubated for > 10 min, or for 3T3 cells expressing mCherry-GFP-LC3, there were no further additions prior to imaging. Tonicity was maintained by substitution of KCl, MgCl_2_, CaCl_2_, and glucose with equivalent molar NaCl, by substitution of NaCl with choline-Cl^-^, and by substitution of Cl^-^ salts with Na-gluconate, K-gluconate, Ca-gluconate, and MgSO_4_. Hypotonic shock was a 1:1 dilution of medium with H_2_O.

### Imaging

Images were acquired on an Olympus iX71 microscope with automated X-Y-Z stage (Applied Scientific Instrumentation), 20X, 0.75 NA or 60x 1.4 NA oil, lenses, CoolSnap HQ cooled CCD camera (Photometrics), DG-4 xenon lamp (Sutter Instrument Co., Navoto, CA) with automated switching between filters appropriate for Dapi, FITC, Rhodamine, and Cy5 fluorescence as well as bright field channels, and run by Metamorph Software (Molecular Devices LLC, Sunnyvale, CA). Autofocus mode was used to capture quantitative images.

### Propidium iodide, Lysotracker, TMRE, DiBAC4(3), endocytosis, and microtubule staining

For lysosome and mitochondria staining, cells were exposed to 10 μM Hoechst for 5 min, washed 5x, and incubated with live medium +/- K^+^ for 90 min. Lysotracker Red DN99 (70 nM) or TMRE (5 nM or 30 nM) or DIBAC4(3) (0.5 or 2 μM) Anaspec #AS-84700) was then added for > 10 min followed by imaging. For endocytosis, cells were treated as above and then incubated with FL-EGF (1.43 μg/mL) for 20 min, washed 3x in live cell medium (+/- K^+^) and then imaged. Other cells were exposed to FL-EGF, Hoechst, washed 5x and incubated +/- K^+^ for 90 min as above and then stained with 2 μM DIBAC4(3) and 2 μM propidium iodide and imaged and then incubated an additional 18 h in a humid environment at 37°C and then imaged. For microtubule staining, cells were treated as above and then fixed in 100% methanol at -20°C for 5 min, aspirated, washed 3x with TBS-Tween (150 mM NaCl, 20 mM Tris, 0.1% tween, pH 7.5), and incubated with 1:167 dilution Cy3 anti-tubulin antibody (Sigma Cat # C4585) in TBS-tween for 30 min, washed 5X in TBS-tween and imaged.

### Image analysis

**Fig 1:**
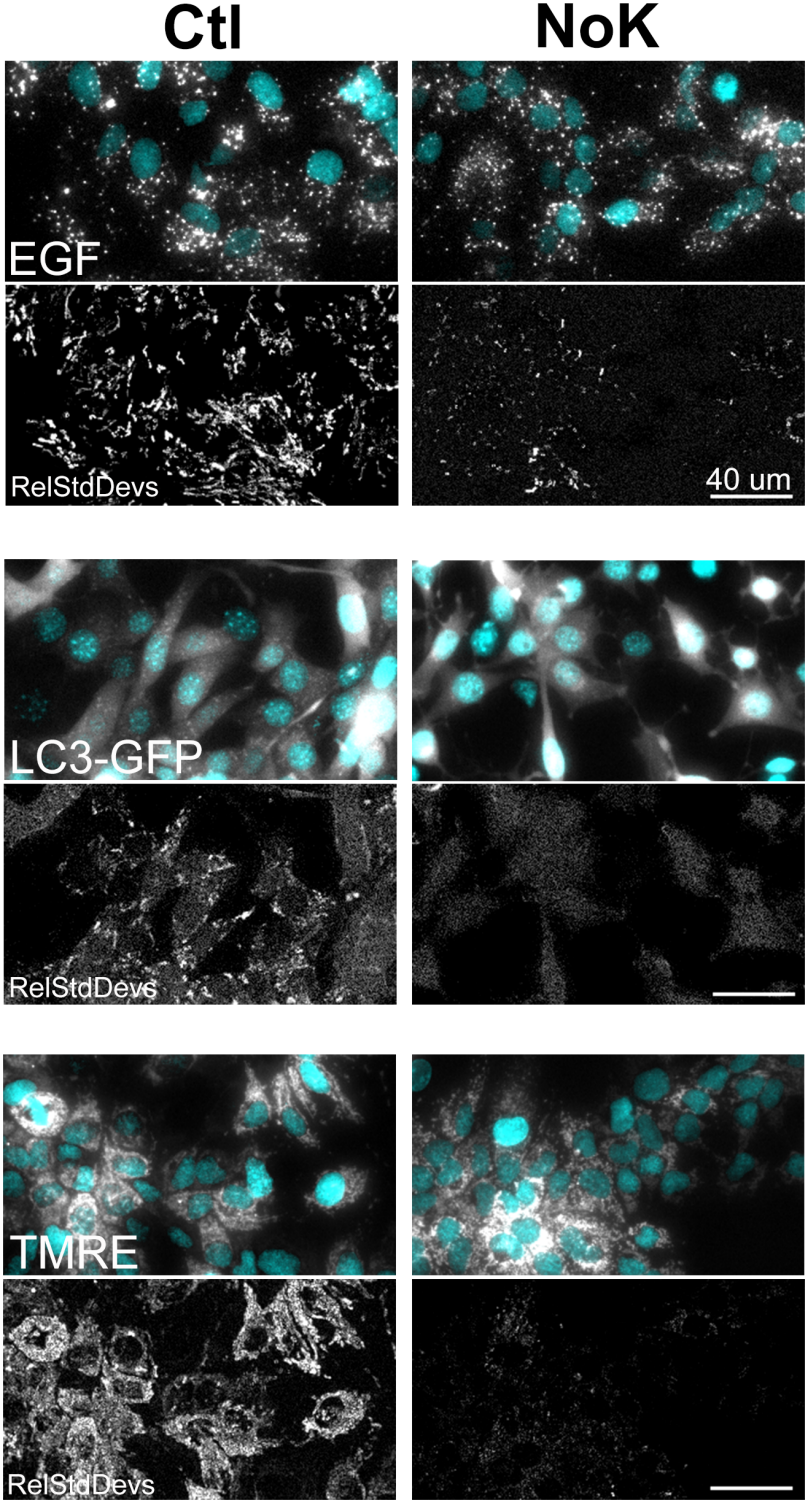
Reduced movement of endocytic organelles, autophagosomes, and mitochondria in cells exposed to potassium free medium. **(Top)** Huh-7 cells were exposed to 1.43 μg/mL FL-EGF (white) for 10 min, 10 μM Hoechst nuclear stain (cyan) for 5 min, washed and incubated 120 min medium +/- K^+^, and imaged by time lapse fluorescence microscopy. Representative fields of cells, cropped to 320x180 pixels from the original 696x520, show internalized EGF (EGF) and below these the relative standard deviations (RelStdDevs) of the pixel intensities of the movies of the same fields are shown where streaks indicate organelle movement. As can be seen, cells in medium lacking K^+^ (NoK) look similar to control (Ctl) in static images but show many fewer streaks in the RelStdDev image, indicating less movement. **(Middle)** 3T3 cells that had been stably transfected with mCherry-GFP-LC3, a marker of autophagosomes, were incubated in Hoechst, and washed and incubated in medium for 90 min and imaged and displayed as for FL-EGF. Autophagosomes (GFP-LC3) also showed strongly reduced motility under K^+^ depletion (NoK), indicated by fewer streaks and lower brightness of the RelStdDev image. **(Bottom)** Huh-7 were incubated with Hoechst, washed and incubated +/- K^+^ for 90 min, and exposed to 30 nM TMRE, and imaged and displayed as for the others. Again, TMRE appears similar in static Ctl and NoK conditions, but movement is strongly reduced in the absence of K^+^. Left and right images are displayed with the same brightness settings to reflect the quantitative difference in appearance and movement.

Images were quantified using macro programs written for ImageJ (National Institutes of Health, Bethesda, Maryland, http://rsb.info.nih, [24]). Movement of FL-EGF, Lysotracker, LC3-GFP, and TMRE was quantified as the normalized mean of the standard deviations of fluorescence intensity of each pixel during the 1 min time lapse movie and labeled as, “Background Subtracted Mean of the Relative Standard Deviations of the Movie Pixel Intensities, % of Control”. Specifically, images containing 696x520 pixels of fluorescence signal were captured every 2 sec for 1 min to create each movie. The mean brightness of all images was normalized to 100 and a Z projection of the movie was created using the ImageJ standard deviation method. This places the standard deviation of each pixel of the movie at the pixel location. These values were then divided by a Z projection of the same movie using the ImageJ average intensity method, which places the average value of the movie at each pixel location. This division yields an image consisting of the relative standard deviations of each pixel, and labeled “RelStdDevs”. Examples of such images are shown in Fig 1, cropped to 320x180 pixels for clarity. Normalizing this way reduced effects of differing image and organelle brightness. The mean value of the image was then subtracted by the mode value to increase sensitivity, a method of background subtraction. This value was then divided by the number of cells per movie to give the amount of movement per cell. Finally, this was normalized to the mean value for all control movies for each experiment. This gave the value that is presented in the figures and Table 1 (i.e. Background Subtracted Mean of the Relative Standard Deviations of the Movie Pixel Intensities, % of Control). Controls and experimental treatments were run side-by-side and movie acquisitions were alternated between all conditions. The number of cells per movie was taken as the number of nuclei and these were counted by segmenting (digitizing) images of Hoechst nuclear stain by applying Gaussian blur and spot enhancing [25] filters followed by auto threshold using the ImageJ Triangle method, followed by analyze particles command using appropriate min max and circularity set points. Single data points of motility represent entire image fields, which contained an average of 189 cells. Other data (e.g. Table 1) represent at least 5 fields with 2–3 experiments per condition unless indicated. To quantify fluorescence per cell, nuclei were segmented as above and a "doughnut" region was created by enlarging the nucleus selection by 3 μm in x and y dimensions and selecting the region between this and the nucleus. The digital outlines were overlaid to the fluorescence channels DiBAC4(3) (FITC), Lysotracker Red DN99 (Rhodamine), TMRE (Rhodamine), Alexa 647 EGF (Cy5), Cy3 anti-tubulin (Rhodamine) and mean intensity was measured. Numbers reported are for > 1000 cells with 2–3 experiments for all conditions. Propidium iodide positive (i.e. non viable) was scored as > 200 mean intensity within the nucleus as mean intensity subtracted from the image mode. The mean intensity and standard deviation are reported for individual cells, unmodified in relative fluorescent units of the 12 bit (0–4095) CoolSnap HQ camera.

**Table 1:** Effects of potassium free medium on different cell lines.

Cell Line	% Change in EGF, LC3, or LysotrackerMotility	% Change in Mitochondrial TMRE Motility	% Change in TMRE Intensity	% Change in DiBAC Intensity	Cell Retraction
Huh7	**-68.8** (EGF) (100.00±16, 31.18±9)	**-88.9** (ns) (100.00±nd, 11.10±nd)	**+14.1** (1507±322, 1719±470)	**-20.9** (1174±354, 928±229)	No
3T3	**-35.7** (LC3-GFP) (100.00±29, 64.28±20)	n/a	n/a	n/a	Yes
HeLa	**-46.2** (LysoTr) (100.00±13, 53.82±11)	**-73.1** (100.00±3.96, 26.87±4.23)	**+8.4** (479.1±72, 519.5±83)	**+40.2** (339.2± 46, 475.5± 92)	No
MDCK	**-78.4** (LysoTr) (100.00±38, 21.64±6)	**-64.5** (100.00±38, 35.46±39)	**+12.3** (582.7±1070, 654.1±1150)	**-8.0** (797.3±173, 733.8±173)	Yes
293FT	**-61.1** (LysoTr) (100.00±35, 38.94±14)	**-81.2** (100.00±12, 18.78±8)	**+11.1** (522.1±1380, 580.3±1480)	**+20.6** (563.2±101, 679.2±152)	No

Parentheses values are average movement, %, or average fluorescence, arbitrary units, ± SD.

n/a: not available, cells contain LC3-GFP-mCherry fluorescence in red and green channels.

All values are p<0.01 vs control medium except for ns, not significant (single measurement), nd, not done.

### Mitochondria and glycolytic stress tests

Cells were seeded onto XFe96 FluxPak 96 well plates at 70% confluence in DMEM supplemented medium and the following day washed 5X with 240 uL control or K^+^ free assay buffers that were based on the Agilent base medium. Glycolysis stress test assay buffer (lacking glucose) was 145 mM NaCl, 1 mM MgCl_2_, 1.8 mM CaCl_2_, 0.91 mM NaH_2_PO_4_, 2 mM glutamine, pH 7.4 with NaOH. Mitochondria stress test assay buffer additionally contained 10 mM glucose, 1 mM Na pyruvate. KCl (2.5 mM) was included in the control experiments. Cells were allowed to equilibrate for 90 min at 37°C in the absence of CO_2_, and 20 uL of drugs were loaded into the XF96 4 port injection cartridge to achieve the final concentrations indicated. The Seahorse XF analyzer system (Agilent Technologies, Inc, Santa Clara CA) was allowed to run for the times indicated and drugs were automatically injected and mixed and oxygen consumption and extracellular acidification was read according to an automated protocol. Data was read into Wave Desktop software and exported to Microsoft Excel. No normalization or other alterations were applied. Cell density was carefully manipulated and drug concentrations were calibrated in preliminary experiments.

### Isolation of ASOR-labeled endocytic vesicles

Vesicles containing endocytic fluorescent asialoorosomucoid (ASOR) were isolated from rat livers using a method similar to that previously described [26] with modifications indicated below. All animal procedures were approved by the University Animal Use Committee. Fifty micrograms of Texas Red labeled ASOR was injected into portal veins of ketamine and xylazine anesthetized 200- to 250-g male Sprague Dawley rats (Taconic Farms, Germantown, NY, USA). After 5 min, a time when internalized ligand is found in early endocytic vesicles [27], animals were sacrificed by exsanguination under fully unconscious anesthesia and livers were blanched with cold PBS, removed, and homogenized with 20 strokes in a loose dounce in 15 mL of MEPS (5 mM MgCl_2_, 5 mM EGTA, 35 mM K_2_-Pipes, 0.25 M sucrose, pH 7.4) plus 4 mM DTT, 1:20 protease inhibitor (Sigma P8340), 1 mM phenylmethanesulfonyl fluoride (PMSF). This was centrifuged 2500 x g for 10 min to create a post nuclear supernatant and protease inhibitors and DTT were re-added. The supernatant was brought to 50% w/v Nycodenz (Accurate Chemical and Scientific Corporation, AN7050) using an 80% stock solution and loaded into the bottom of a density gradient containing 50, 24.1, 17.2, 0% Nycocodenz in MEPS plus 4 mM DTT and centrifuged at 260,000 x g (39,000 rpm) for 2 h in a Beckman SW41 rotor. The cloudy interface between 0 and 17.2% Nycodenz was found to contain the endocytic vesicles containing fluorescent ligand, and this was collected and stored as single use aliquots at -80°C.

### In vitro motility assays

Full brightness rhodamine tubulin (Cytoskeleton Inc, # TL590M) and unlabeled tubulin (Cytoskeleton Inc, # T240) were mixed to achieve 1:20 labeled: unlabeled stock of rhodamine tubulin and clarified by 10 min centrifugation at 200,00 x g (14,000 rpm) and frozen as 4 uL aliquots. Aliquots were thawed and polymerized by heating to 37°C for 5 min, sheared by pipetting up and down, and added to warm 4 uL BRB80 buffer (80 mM Pipes-K_2_, 1 mM EGTA, 1 mM MgCl_2_) plus 1 mM GTP along with a second 4 uL aliquot of rhodamine tubulin. This was further polymerized at 37°C for 6 min and 100 uL of microtubule buffer (BRB80 plus 1 mM GTP plus 20 μM Taxol) was added followed by centrifugation for 1 min in a Beckman Airfuge at 10 psi. The pink pellet was resuspended in 140 uL microtubule buffer. For the assays, microtubules were diluted 1:25 in microtubule buffer and added to optical chambers that were coated with 30 μg/mL DEAE dextran [26]. After 3 min chambers were washed 1x in blocking buffer (35 mM Pipes-K_2_, 5 mM MgCl_2_, 1 mM EGTA, 0.5 mM EDTA, 4 mM DTT, 20 μM Taxol, 2 mg/mL BSA, 5 mg/mL casein, pH 7.4) and placed on ice. Vesicles were thawed, diluted 1:3 in blocking buffer, and added to the chambers and incubated for 15 min. These were washed 1x in assay buffer (70 mM NaCl, 5 mM MgCl_2_, 5 mM KCl, 20 mM Hepes-Tris (12.5 mM Hepes, 7.5 mM Tris), 4 mM DTT, 10 μM Ouabain, 20 μM Taxol, pH 7.4) or alternate solute substituted buffers, and stored for up to one hour on ice. Chambers were serially removed from ice, warmed to room temperature (1 min), placed under the microscope at 35°C, and multi-channel fluorescence image capture was initiated at 1 frame per 2 sec for 46 frames along with addition 40 uL of assay buffer or solute substituted buffer containing 100 μM ATP. As for live cell motility, controls were performed alongside experimentals and rotated through the different conditions so that chambers containing the same condition were not assayed consecutively.

## Results

### Organelle motility is reduced by short term elimination of extra cellular potassium

To investigate the movement of organelles, a robust live cell motility assay was developed. We found that fluorescent EGF (FL-EGF) produced very strong intracellular fluorescence when taken up by endocytosis into Huh-7 cells. Cells were exposed to Alexa 647 labeled EGF for 10 min at 37°C followed by Hoechst nuclear stain for 5 min and then extensively washed in live cell medium (120.8 mM NaCl, 2.5 mM KCl, 1 mM MgCl_2_, 1.8 mM CaCl_2_, 25 mM Glucose, 20 mM Hepes-Tris (12.5 mM Hepes, 7.5 mM Tris), pH 7.4). Cells were then incubated for 15–180 min at 37°C, and imaged at 1 frame / 2 sec for 1 min, generating 30 frames of the moving endosome and lysosome array. Huh-7 cells, like many cultured cells, express high EGF receptor levels and the endocytic fluorescence is strongly above background and present in nearly every cell. We typically used 1.43 μg/mL FL-EGF, which is in the range recommended by the manufacturer. This will have a molar concentration equivalent of 65–130 ng/mL unconjugated EGF, since the molecular weight of alexa fluor streptavidin is 11–22 fold greater than that of EGF alone, with the variability due to the presence of potentially more than one biotin-streptavidin per EGF (technical support, Thermo Fisher Scientific). We used several strategies to quantify the movement including, tracking individual vesicles, measuring the frame to frame change in area occupied by fluorescence, and measuring the relative standard deviation of the intensity changes of each pixel during the movie. The mean of the relative standard deviations of the movie pixels proved to be the simplest and most sensitive indicator of the movement of EGF and other fluorescent markers. This was expressed as percent of control and labeled as, “Movement of FL-EGF (Background Subtracted Mean of the Relative Standard Deviations of the Movie Pixel Intensities, % of Control)".

To test the effect of K^+^ depletion on vesicle movement, Huh-7 cells were exposed to FL-EGF following the protocol above and then incubated > 60 min in live cell medium or medium where KCl was substituted with NaCl. Fig 1 (top panels) shows microscope images of FL-EGF and cell nuclei cropped to 320x180 pixels from the original 696x520, and beneath these are images of the relative standard deviations (RelStdDevs) of the movies of the same field. In the static images, control (Ctl) and K^+^ depleted (NoK) Huh-7 cells looked quite similar and did not exhibit different FL-EGF intensity (note that cells underwent endocytosis prior to K^+^ depletion). Visualizing by the relative standard deviation method, control cells were full of white streaks resulting from vigorous back and forth and long distance movement of the FL-EGF vesicles whereas K^+^ depleted cells show only small spots and occasional streaks. The effect is even more evident in S1 and S2 Movies of these fields. In many K^+^ depleted cells, movement appears completely blocked whereas in control conditions all cells exhibit back and forth vesicle movement, although there is considerable cell to cell variation in the amount of movement. Interestingly some residual vesicle movement remains in the K^+^-depleted cells. We never observed complete arrest of movement that is seen in when cells are placed into fixative or killed by other means.

To determine if this effect of K^+^ depletion was specific to EGF-labeled organelles or cell type, we repeated the imaging experiments for 3T3 cells that had been stably transfected with mCherry-GFP-LC3, a marker for autophagosomes, and for Huh-7 cells that had been treated with the mitochondrial staining dye, TMRE. As can be seen in Fig 1 and in S3–S6 Movies, in all cases motility was strongly reduced by exposure to K^+^-free medium. This suggests a global decrease in organelle movement that is independent of any effects of EGF or EGF signaling since the TMRE and LC3-EGF experiments did not involve treatment with EGF. White streaks are seen for LC3 and TMRE in the relative standard deviation image projection of control cells, whereas 'NoK' treated cells showed mainly haziness resulting from short, vibrational movement that remained (the brightness settings were set identically). 3T3 cells underwent some contraction of their cell bodies during K^+^ depletion (S1 Fig), but this did not prevent measurement of autophagosome movement, which was clearly reduced. Also apparent from these images is the strength of FL-EGF signal, which allowed robust visualization and quantification compared to the crowded mitochondria (TMRE) or the often diffuse (i.e. soluble) GFP-LC3.

**Fig 2:**
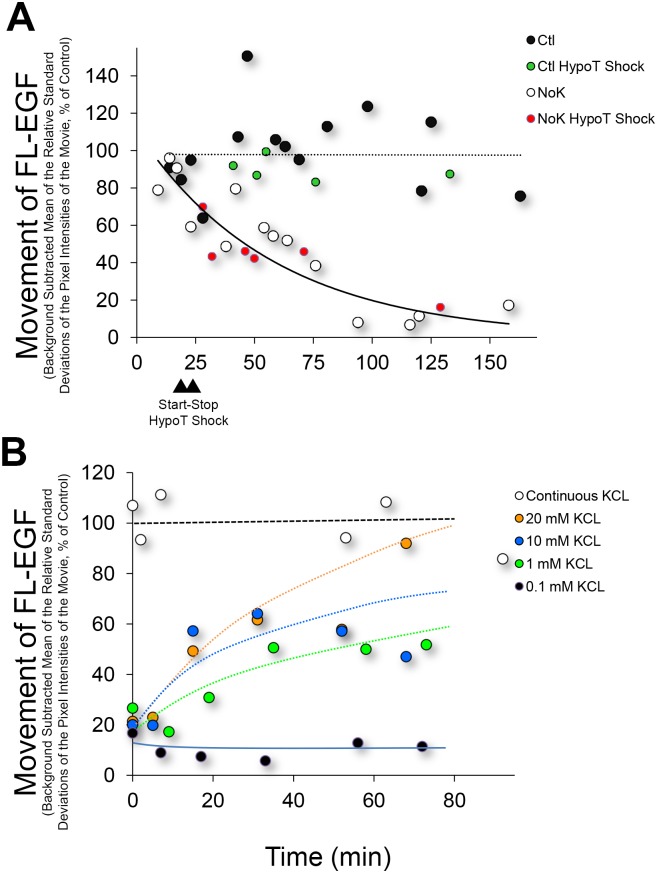
Time course for inhibition of FL-EGF movement and rescue of inhibition of movement by addition of KCl. (**A)** Huh-7 cells were exposed to FL-EGF, Hoechst, and washed and incubated in control (Ctl, black circles) or K^+^ free (NoK, white circles) live medium, and imaged at the times indicated. FL-EGF movement was quantified by measuring the mean of the relative standard deviations of the movie pixel intensities and normalizing to the mean value of the controls. Each circle represents a field of cells containing on average 143 nuclei for these experiments. Movement of FL-EGF containing vesicles is seen to decrease through approximately 90 min, whereupon it plateaus near 10% of control. Exposure of cells to hypotonic shock for 5 min (stop and start times indicated by triangles) did not appear to affect the time required to reduce movement under K+ free medium (red circles) nor affect control movement (green circles) following re-addition of isotonic medium. (**B**) Cells were exposed to Ctl (white circles) or NoK (black and colored circles) medium for 90 min followed by addition of KCl at the concentration indicated.

To determine the scale of the effect, we imaged FL-EGF organelles as a function of time after incubation in K^+^ free medium. The amount of organelle movement was expressed numerically by plotting mean of the relative standard deviations of fluorescence intensity of each pixel during the 1 min time lapse movie, dividing by the number of nuclei per field, and then expressing as percentage of the value obtained in the control experiments. As can be seen in Fig 2, movement decreased steadily through approximately 90 min in the absence of K^+^, whereas movement under control conditions remained constant. Each dot in Fig 2 represents a single movie containing on average, 143 cells for these experiments. For all experiments, control studies were performed alongside experimental studies on the same day and with the same treatment duration. Significant variability in the amount of movement was observed for both control and K^+^ depleted cells. Individual cells or groups of cells often show quite high or low motility for unknown reasons. Regardless of this variability, the trend was clear, motility decreased through 90 min and after this remained at 5–20% of control motility but did not drop to zero over the 160+ min of monitoring. Larkin et al [17] found that they could reduce the time required for K^+^ free medium to inhibit endocytosis by introducing a 5 min hypotonic shock prior to exposure to K^+^ free medium. Hypotonic shock should promote cell swelling and the loss of K^+^. However, as seen in Fig 2A (red circles), a 50% hypotonic shock did not noticeably affect the rate that organelle motility decreased following exposure to K^+^ free medium (compare red circles to white circles). Hypotonic shock also did not appear to affect organelle movement when cells were placed back into standard medium (compare green circles to black circles). To determine whether the reduction in organelle motility could be rescued by addition of K^+^, we placed cells in K^+^ free medium for 90 min and then added KCl back to the medium. As can be seen in Fig 2B, 0.1 mM KCl was unable to rescue motility (black circles). However addition of 1, 10, or 20 mM KCl increased motility in a concentration dependent manner. The rescue of motility was not instantaneous, as can be seen with addition of 20 mM KCl, where 60 min was required for motility to approach control levels.

### Sodium and chloride depletion also inhibit organelle motility

**Fig 3:**
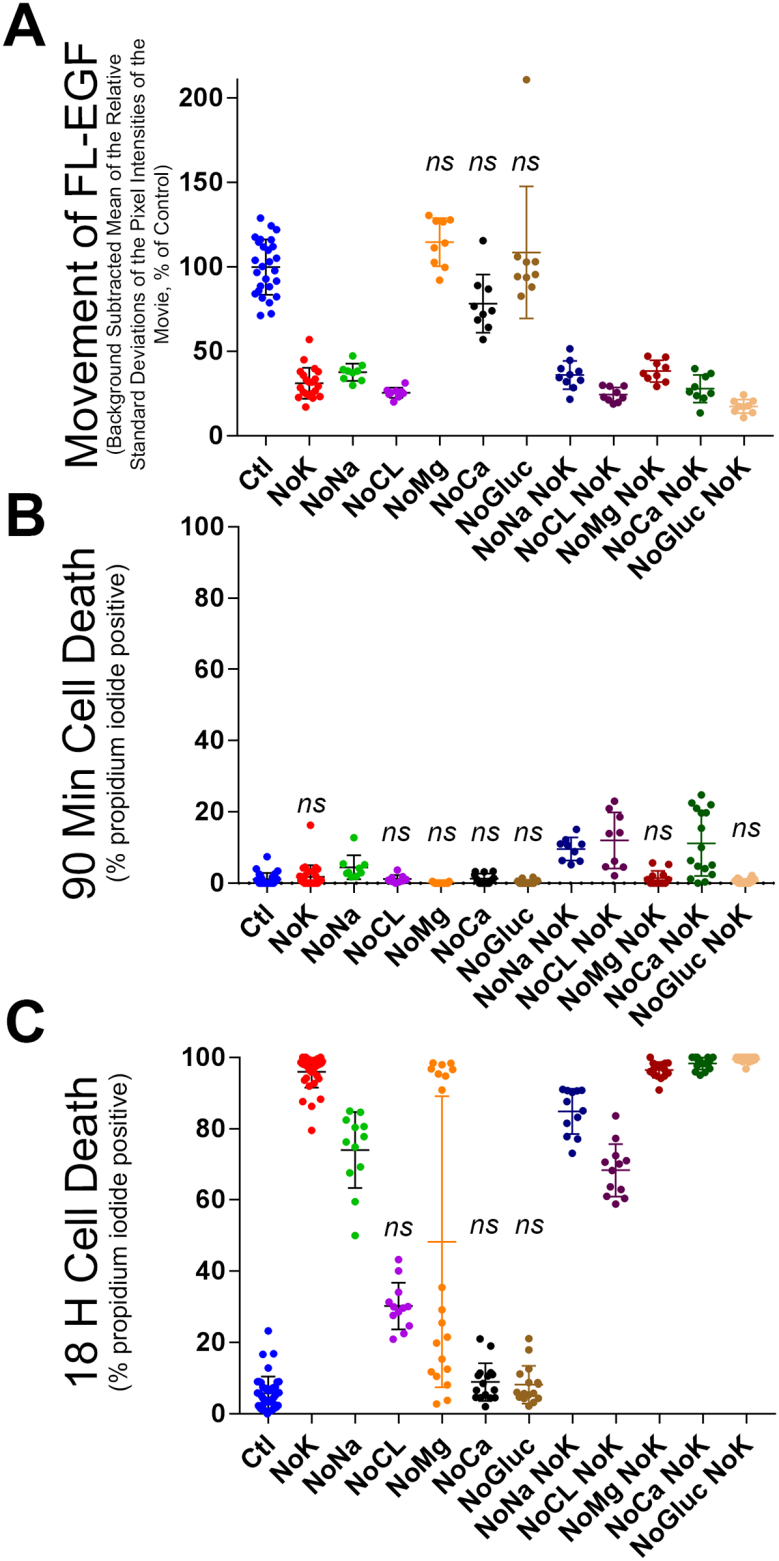
Effect of solute substitutions of the medium on the movement of internalized FL-EGF and cell viability. **(A)** Cells were exposed to FL-EGF and Hoechst and then washed and incubated with various media for 90 min. Movement of internalized FL-EGF was imaged and then quantified as in Fig 2. Elimination of potassium (NoK), sodium (NoNa), chloride (NoCL), magnesium (NoMg), calcium (NoCa), glucose (NoGluc) and elimination of both sodium and potassium (NoNa NoK) etc., is as indicated. (**B)** Cells treated as above were additionally scored for cell death by co-staining of Hoechst stained nuclei with 2 μM propidium iodide. Cells were predominantly viable after 90 min for all treatments. (**C)** Cells from (B) were incubated for 18 hours and imaged and quantified for cell death. After 18 h, K^+^ depletion as well as other conditions led to wide-spread death. Bars show mean +/- SD. Statistics were one way ANOVA followed by Kruskal-Wallis analysis of variance, (*ns*), not significantly different from control.

We considered whether other components of the extracellular medium are also critical for maintaining organelle motility. To answer this, cells were allowed to take up FL-EGF and Hoechst nuclear stain, and then washed in medium lacking different chemical components of the base medium. This was followed by a 90 min incubation at 37°C and image capture and quantification as above. Tonicity was maintained by substitution of KCl, MgCl_2_, CaCl_2_, and glucose with equivalent solute species of NaCl, by substitution of NaCl with choline-Cl^-^, and by substitution of Cl^-^ salts with Na-gluconate, K-gluconate, Ca-gluconate, and MgSO_4_. It was seen that elimination of K^+^, Na^+^, and Cl^-^ all diminished FL-EGF vesicle motility (ANOVA, and p < 0.01 by Tukey HSD) whereas elimination of magnesium and glucose had no significant effect, and elimination of Ca^2+^ showed a moderate reduction in motility (ANOVA, and p < 0.05 by Tukey HSD, Fig 3A). To determine if the effect of K^+^ removal might require the presence of other ions, we performed double depletions. As can be seen in Fig 3A, double depletions of K^+^ and any one of the other solutes did not affect the outcome; motility continued to be strongly reduced. For instance, removal of either Na^+^ or K^+^ led to 60–70% reduction in motility while removal of both ions also led to ~70% reduction. As with K^+^ deletion alone, motility was never completely eliminated and occasional cells showed high motility. It should be noted that Fig 3A looks only at the motility of FL-EGF and therefore the effects of depletion of these other solutes may be specific for EGF or EGF signaling.

We did not specifically look for changes in cell shape or organelle distribution, but such changes might be expected. Indeed, Ca^2+^ depletion led to contraction or rounding up of the cytoplasm and the endosome and lysosome array (S2 Fig). Despite this contraction (i.e. despite increased focus of FL-EGF at the cell center) organelle movement was still very active under extracellular Ca^2+^ depletion as seen by eye and as measured by the standard deviations of movie pixel intensities (Fig 3A). It is possible that the small decrease in motility for Ca^2+^ depletion was in fact due to cell contraction. For media lacking both Ca^2+^ and K^+^, cells showed less contraction, yet motility was strongly reduced. Cl^-^ depletion of the medium also led to moderate cell contraction, and in this case, organelle motility was clearly reduced as compared to control. Moderate cell contraction was also observed when medium was depleted of Cl^-^ and K^+^. We did not detect clear differences in cell shape or FL-EGF distribution for the other treatments, although more extensive analyses may be required.

It may be questioned how much damage the cells incur from the solute substitutions. We quantified toxicity by measuring propidium iodide staining of cell nuclei. Propidium iodide is excluded from living cells but stains dead cell nuclei brightly. We created an ImageJ macro program to score the number of Hoechst staining nuclei that co-stained for propidium iodide. Initial studies indicated that cells were viable after 90 min, and we therefore additionally measured cell death after 18 h. Note that these media were not designed for long term cell culture, for instance CO_2_-HCO_3_ buffering was avoided. As can be seen in Fig 3B and 3C, most treatments did not increase cell death at 90 min, although it was somewhat increased over control (1.06%) following double depletion of K^+^ and either Na^+^ (9.64%), Cl^-^ (12.04%), or Ca^2+^ (11.23%). After 18 h however, K^+^ or Na^+^ depletion led to wide-spread cell death, and K^+^ depletion combined with magnesium, Ca^2+^, or glucose depletion resulted in nearly 100% cell death. Double depletion of K^+^ and Na^+^ or Cl^-^, showed slightly lower cell death than K^+^ alone. Overall these results confirm what might be expected and as has been understood for some time [21], that cultured cells require K^+^ and Na^+^ for long-term survival. Removal of these components for extended periods is a toxic event. It is expected that removal of Cl^-^, magnesium, or Ca^2+^ would also cause cell death with longer incubation. However at early time points when organelle motility is inhibited, the cell populations did not show loss of viability. The loss of organelle motility cannot be attributed to cell death per se. Instead, removal of K^+^ or Na^+^ from the medium produces changes that reduce organelle movement and subsequently lead to cell death.

### Under potassium depletion the membrane potential of lysosomes and mitochondria is maintained as is the microtubule cytoskeleton

**Fig 4:**
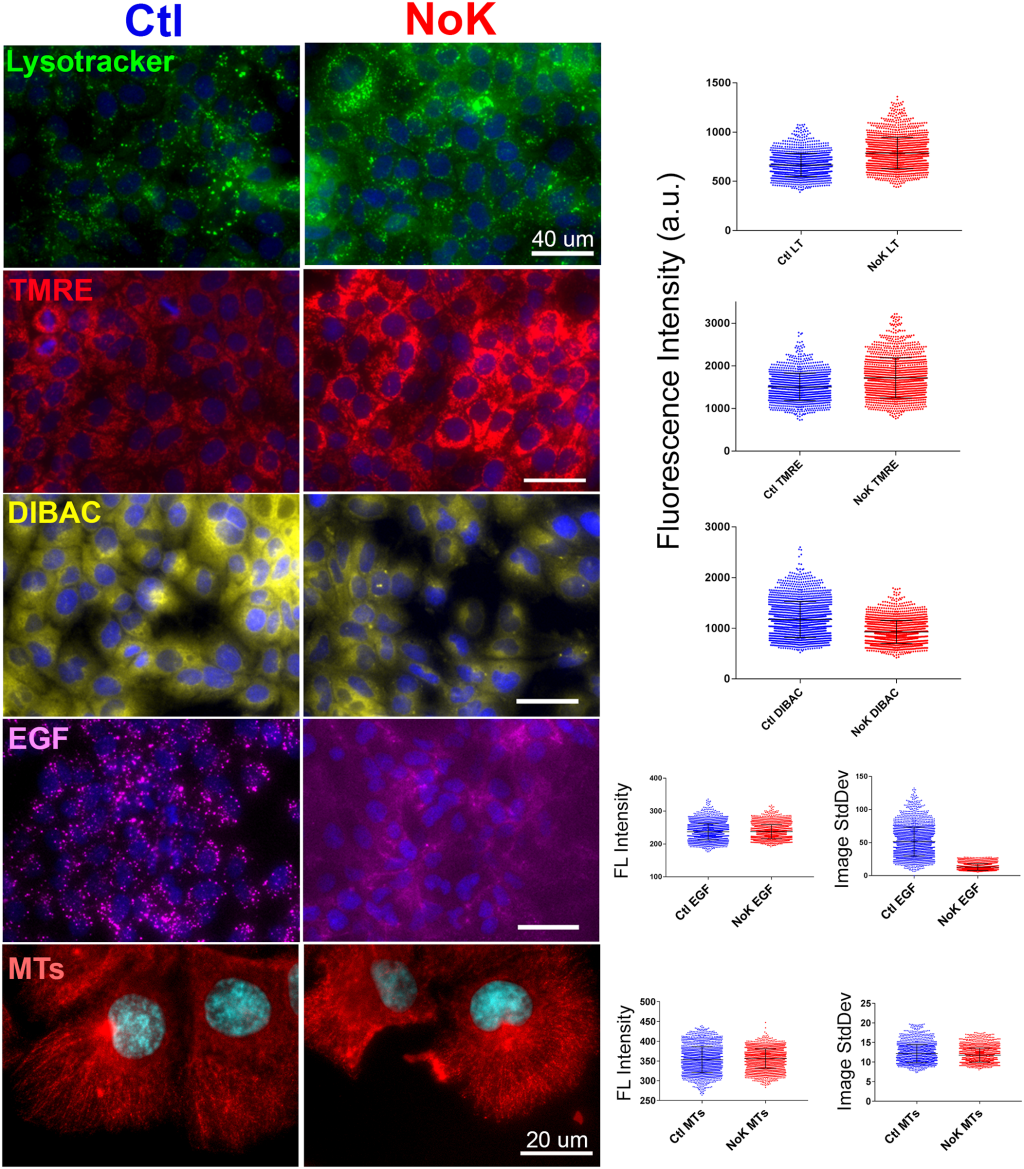
Organelles and microtubules appear normal under potassium depletion while endocytosis is inhibited. Cells were exposed to Hoechst nuclear stain and washed and incubated +/- K^+^ for 90 min followed by addition of 70 nM Lysotracker, 30 nM TMRE, or 1.43 μg/mL FL-EGF, as indicated. For other cells, FL-EGF was first added, followed by Hoechst, and then the cells were incubated 90 min +/- K^+^, followed by addition of 2 μM DIBAC4(3) or fixation with methanol and staining for microtubules (MTs), as indicated. Example images are presented along with measurement of mean cytosolic fluorescence intensity +/- SD (right). Each dot represents a single cell. At the bottom right, mean cytosolic fluorescence intensity +/- SD as well as mean standard deviation +/- SD (Image StdDev) is presented per cell. Image StdDev quantifies the variation in intensity across the cells and is strongly reduced under K^+^ depletion for FL-EGF, indicating that endocytosis is inhibited.

One explanation for reduced organelle motility in K^+^ depleted cells would be a loss of ATP and ATP-dependent activities. We employed a series of fluorescent dyes to determine whether other ATP-dependent processes are affected. Lysotracker is a weak base that concentrates in lysosomes and other acidic organelles that contain active V-ATPases. TMRE is a cationic dye that concentrates in the matrix of mitochondria in proportion to inner membrane potential [28]. Cells were treated with K^+^ free medium for 90 min followed by exposure to the dyes to determine whether organelle membrane potentials are maintained. As can be seen (Fig 4), Lysotracker and TMRE accumulated in organelles for both control and K^+^ depleted cells, displaying their characteristic lysosomal and mitochondria appearance under both conditions. The intensity of Lysotracker and TMRE appeared somewhat stronger in K^+^ depleted cells, and we developed ImageJ macros to quantify cytosolic fluorescence per cell by selecting the Hoechst stained nuclei and measuring the intensity in a region just outside of the nucleus. Intensities of multiple experiments are plotted as column scatter graphs to the right of the representative images in Fig 4. Lysotracker (670.4 +/- 129.3 vs 791.4 +/- 167.1, mean +/- SD, arbitrary fluorescence units, p<0.01) and TMRE (1507 +/- 321.60 vs 1719 +/- 469.80, p<0.01) staining was 14–18% higher for Huh-7 cells exposed to K^+^ free medium. To assess plasma membrane potential, we employed DiBAC4(3), a negatively charged dye that accumulates into cells in inverse proportion to plasma membrane potential; cells with low membrane potential (e.g. dead cells) will show bright staining of DiBAC4(3). As seen in Fig 4, Huh-7 cells exposed to K^+^ free medium showed a 20.9% decrease in DiBAC4(3) staining (1174 +/- 354.4 vs 928.3 +/- 228.7, p<0.01), suggesting hyperpolarization of the plasma membrane for these cells. Hyperpolarization may be expected if K^+^ were to leak from the cells under conditions where membrane polarity could not otherwise dissipate [29]. Larkin et al [17] and others have found that K^+^ depletion results in inhibition of endocytosis of EGF and other ligands. To examine whether endocytic inhibition is seen under our conditions, cells were exposed to Hoechst nuclear stain, washed, and treated with medium +/- K^+^ for 90 min, and then exposed to FL-EGF for 20 min, followed by washing in the same medium. In control cells, this resulted in bright fluorescent spots throughout the cytoplasm, as was seen previously (Fig 1). In contrast for K^+^ depleted cells, the distribution of FL-EGF was primarily diffuse with some increased intensity at cell-cell junctions and occasional spots of fluorescence (Fig 4). The sporadic appearance of FL-EGF puncta was reminiscent of the sporadic motility seen under K^+^ depletion and suggests that some cells can resist the treatment. Although the FL-EGF distribution in control and K^+^ depleted cells appeared quite different, quantitation revealed near identical mean fluorescence intensity (237.6 +/- 26.5 vs 237.9 +/- 21.8, mean +/- SD), suggesting an unaltered level of FL-EGF binding to the cells. This agrees with Larkin et al [17] who saw normal or increased binding of ligand to cell receptors under K^+^ depletion. Standard deviation of fluorescence across the cells (Image StdDev, Fig 4) on the other hand, was much higher for control cells (51.5 +/- 21.8 vs 13.0 +/- 5.0, mean +/- SD), reflecting their accumulation of endocytic puncta.

Another mechanism that could account for inhibition of organelle movement is a loss of microtubules. Depolymerization of microtubules essentially arrests the directed, non brownian movement of endosomes and lysosomes in cultured cells [6, 7]. Although others have noted that K^+^ depletion does not affect the microtubule cytoskeleton [17], we wanted to confirm and quantify these observations under our conditions. Cells were incubated with Hoechst nuclear stain and washed and incubated in medium +/- K^+^ for 90 min and then fixed with methanol and stained with fluorescent anti-tubulin antibody. As seen in Fig 4, the microtubule cytoskeleton did not appear affected by K^+^ free conditions and quantification did not reveal any obvious differences, with very similar fluorescence intensity (353.4 +/- 33.0 vs 356.2 +/- 24.5) and standard deviation of intensity across the cells (12.14 +/- 2.3 vs 11.76 +/- 1.8). A gross change in the microtubule cytoskeleton therefore does not appear to explain the differences in organelle motility that are seen under K^+^ depletion.

### Potassium free medium reduces organelle motility and increases mitochondrial TMRE staining in all 5 cell lines that were studied

To determine whether different cell lines responded in the same manner to reduced extracellular K^+^, we performed similar experiments described above on 3 additional lines, HeLa, MDCK, and 293FT (Table 1). In all cases, K^+^ free medium led to reduced motility of organelles containing FL-EGF, Lysotracker, or GFP-LC3. The reduction in motility of these organelles compared to control was 60–80% for Huh-7, MDCK, and 239FT, and 46% for HeLa. The differences compared to control were all statistically significant (p<0.01), and they involved analysis of multiple experiments with an average of 13 image fields each containing hundreds of cells. All of the cell lines also showed a 60–90% reduction in TMRE-labeled mitochondria motility in K^+^ free medium as compared to control (p<0.01 for all). Although visualizing individual mitochondria is hindered by their crowded appearance within cells, the standard deviation of the movie pixels method was able to detect large differences in their movement. These measurements demonstrate that the reduction of motility was not specific for FL-EGF containing organelles nor EGF signaling since, except for measurements of EGF itself, the cells of Table 1 were not treated with any form of EGF. We also quantified the cytosolic intensity of TMRE and DIBAC4(3) staining for all of the cell lines. This revealed increased TMRE intensity under K^+^ depletion and either decreased (e.g. 1174 +/- 354, vs 928 +/- 229, for Huh-7 cells,) or increased (e.g. 339.2 +/- 46 vs 475.5 +/- 92, for HeLa) DIBAC4(3) intensity. All of these differences compared to control were highly significant (p<0.01) and involved analysis of thousands of cells and multiple experiments for each condition. Previous studies have shown significant differences in both the rate of loss of K^+^ as well as physiologic response to K^+^-depleting treatments for different cell lines [17, 19], and therefore differences in the change of DIBAC4(3) and TMRE staining was not entirely unexpected. Interestingly, all of the cells showed reduced organelle motility in the absence of K^+^, suggesting that this response may be universal. The strong staining of mitochondria indicates the cells are energetically active. Although it is conceivable that K^+^ depletion could alter accumulation or fluorescence of these dyes in unexpected ways, the evidence suggests that loss of motility correlates with an increased mitochondrial potential but does not correlate with changes in plasma membrane potential, which may be increased or decreased depending on the cell line. We additionally found some degree of contraction of the cytoplasmic area 90 min after removing K^+^ from medium for 3T3 and MDCK cells (S1 Fig). This might relate to a loss of substrate attachments, changes in cytoskeletal based plasma membrane flow, or other processes, but it did not prevent measurement of organelle motility. The standard deviation method readily measures movement of organelles that appear crowded, as seen with the mitochondrial studies. Contraction occurred during the 90 min incubation, not during the short time scale of the time-lapse imaging.

### Mitochondrial function is maintained while extracellular acidification is moderately reduced by potassium depletion

**Fig 5:**
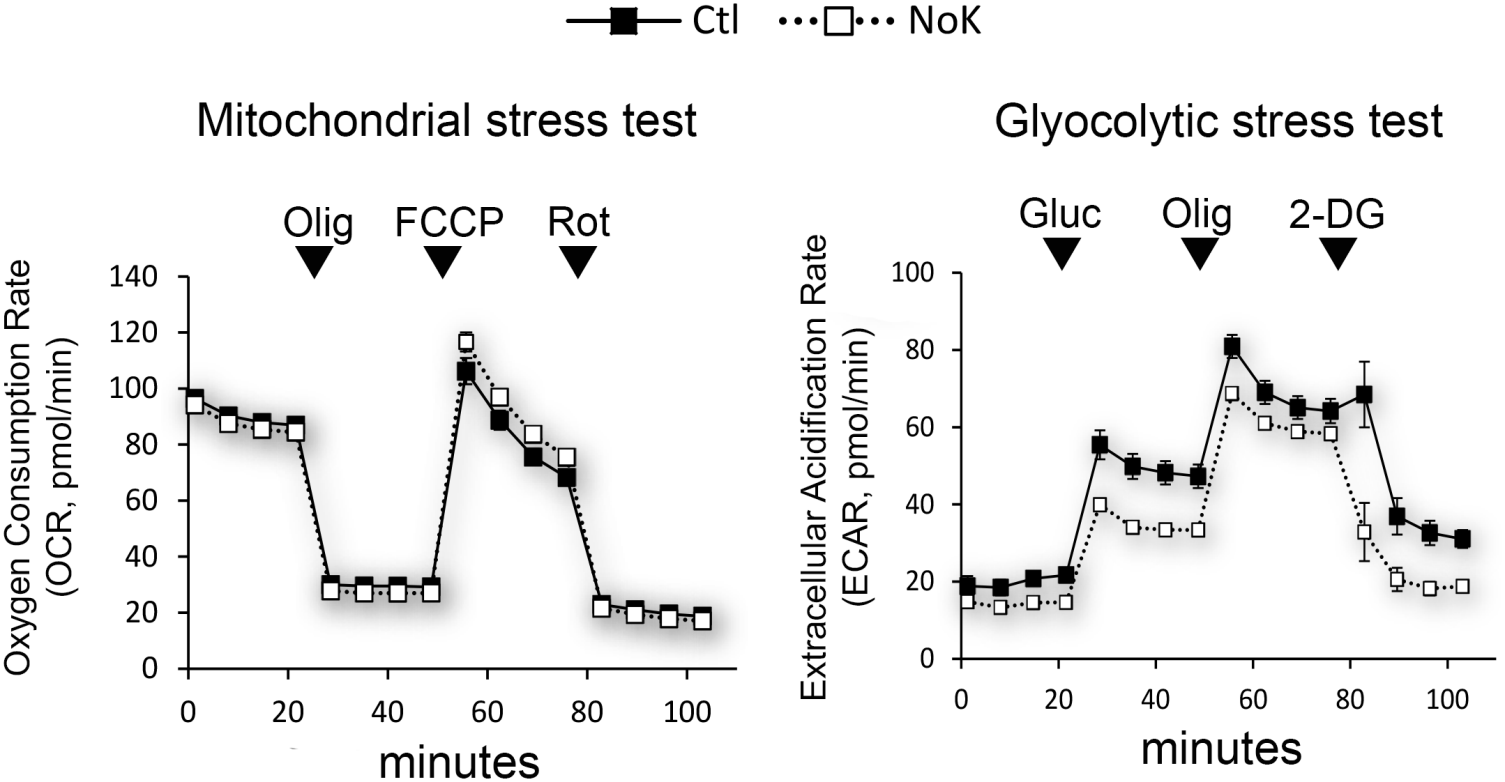
Potassium depletion of Huh-7 cells results in a similar mitochondrial oxygen consumption rate and a moderately decreased extracellular acidification rate. Huh-7 cells were cultured on XFe96 FluxPak multi-well plates and subject to mitochondrial and glycolytic stress tests using a Seahorse XF analyzer system. (**A)** Cells were treated with standard (black squares) or K^+^ free (white squares) medium for > 90 min and assessed for oxygen consumption rate (OCR) under baseline conditions (first 4 points), followed by addition of 1 μM oligomycin (Olig), 2 μM FCCP, 0.5 μM Rotenone (Rot), at the times indicated. Similarly, extracellular acidification was measured under baseline conditions followed by addition of 10 mM glucose (Gluc), 1 μM Olig, and 50 mM 2-deoxyglucose (2-DG). Bars are +/- SEM and are frequently smaller than the symbol. No normalization or adjustment of the data was performed. Similar OCR values and similar response to the drugs under K^+^ free medium indicates that mitochondria are functioning properly, whereas reduced ECAR values but similar response to the drugs suggests either impaired glycolysis or reduced proton efflux.

Since K^+^ depletion could affect many cellular processes, it seemed reasonable that metabolic features of cells would be altered, and we had already measured changes in the accumulation of organelle and cytosol staining dyes (Fig 4, Table 1). We sought to further characterize the effect of K^+^ depletion by measuring mitochondrial and glycolytic activity using a Seahorse XF analyzer system (Agilent Technologies, Inc, Santa Clara CA). This methodology uses highly sensitive oxygen and pH sensors in multi-well plate format to assess cellular metabolic state. Injection of various drugs during sensing allows inference of mitochondrial and glycolytic function. After determining appropriate drug concentrations in preliminary experiments, we followed the mitochondrial and glycolytic 'stress test' experimental paradigms [30]. The left panel of Fig 5 shows a mitochondrial stress test performed for 11 control (black boxes) and 11 K^+^ depleted wells (white boxes). The y-axis depicts oxygen consumption rate (OCR) and reflects mitochondrial oxidative phosphorylation of the cells. Four points of basal OCR were measured, followed by inhibition of mitochondrial ATP synthase with oligomycin. This reduces oxygen consumption in proportion to mitochondrial ATP production. As can be seen in Fig 5, basal OCR did not appear altered by K^+^ depletion (90.36 +/- 9.6 vs 87.84 +/- 9.4, p > 0.05 mean +/- SD, n = 44, 4 time points, 11 wells), and oligomycin led to a large, similar drop in OCR for both conditions. This indicates that mitochondrial ATP generation is not strongly altered by the short term exposure to low K^+^. FCCP (Carbonyl cyanide-4-(trifluoromethoxy)phenylhydrazone) was then added to uncouple mitochondrial oxygen consumption from ATP generation and allow maximal respiration. This was slightly (10%) higher under K^+^ depletion (84.70 +/- 18.4 vs 93.14 +/- 18.1 pmol/min, Ctl vs NoK, mean +/- SD, p = 0.03, n = 44, 4 time points, 11 wells) whereas subsequent addition of rotenone (ROT) poisoned mitochondrial respiration and reduced the rate of oxygen consumption to non-mitochondrial background, which was very similar in both conditions. No normalization was performed and the error bars (SEM) are shown but were generally smaller than the graph symbols, indicating high reproducibility. We confirmed that motility of FL-EGF containing vesicles was inhibited under K^+^ depletion using the Seahorse medium, which contains glutamine and sodium phosphate but no additional buffering agent (S3 Fig). These data suggest that oxygen consumption and mitochondrial respiration are not strongly altered and therefore not responsible for reduced organelle movement.

We performed an additional 'glycolytic stress test' to determine whether glycolysis might be affected by K^+^ depletion. Cellular glycolysis results in acidification of the cytosol due to breakdown of glucose into lactate [30]. Protons are excreted from the cell and can be measured by the XF analyzer. In the test, basal extracellular acidification rate (ECAR) is measured in medium lacking glucose. As can be seen (Fig 5, right panel) baseline ECAR was moderately lower under K^+^ depletion (19.9 +/- 5.8 vs 14.3 +/- 2.4 pmol/min, mean +/- SD Ctl vs NoK, p<0.0001, n = 32, 4 time points, 8 wells). Addition of glucose stimulated acidification in both conditions, indicating that glycolysis is active and can be rapidly increased by its substrate. Absolute acidification rates were slightly lower for K^+^ depleted cells (47.91 +/- 10.3 vs 37.45 +/- 7.6 pmol/min, mean +/- SD Ctl vs NoK, p<0.0001, n = 32, 4 time points, 8 wells). As a third step, oligomycin was added to the cells to inhibit mitochondrial ATPase and thereby stimulate glycolysis [30]. This increased ECAR under both conditions but again the values were lower in K^+^ depleted cells (69.81 +/- 10.6 vs 61.72 +/- 6.0 pmol/min, mean +/- SD Ctl vs NoK, p = 0.0004, n = 32, 4 time points, 8 wells). As a final step, 2-deoxyglucose was added to inhibit glycolysis altogether. This decreased ECAR in both cases but levels were still lower under K^+^ depletion (p<0.0001). The glycolysis stress test therefore revealed a responsive glycolytic metabolism but lower ECAR under all conditions. Overall the data indicate that the mitochondria of Huh-7 cells are not metabolically inhibited by short term K^+^ depletion and that glycolysis also remains active and responsive, although absolute levels of extracellular acidification are reduced by 10–40%. This reduction may reflect reduced glycolysis or reduced excretion of protons, the nature of which requires further investigation. Moderately reduced glycolysis could indicate a moderate reduction in cellular ATP. However, previous studies [19, 31] have found that intracellular ATP is not strongly influenced by short term K+ depletion of cultured cells. In addition it is not clear that moderate reductions in ATP can affect organelle motility. For instance in experiments of Fig 3 we eliminated glucose from the extracellular medium, which will reduce glycolysis as was seen Fig 5, yet this did not decrease organelle motility.

### In vitro motility assays demonstrate that organelle motility machinery is directly affected by potassium and sodium depletion

**Fig 6:**
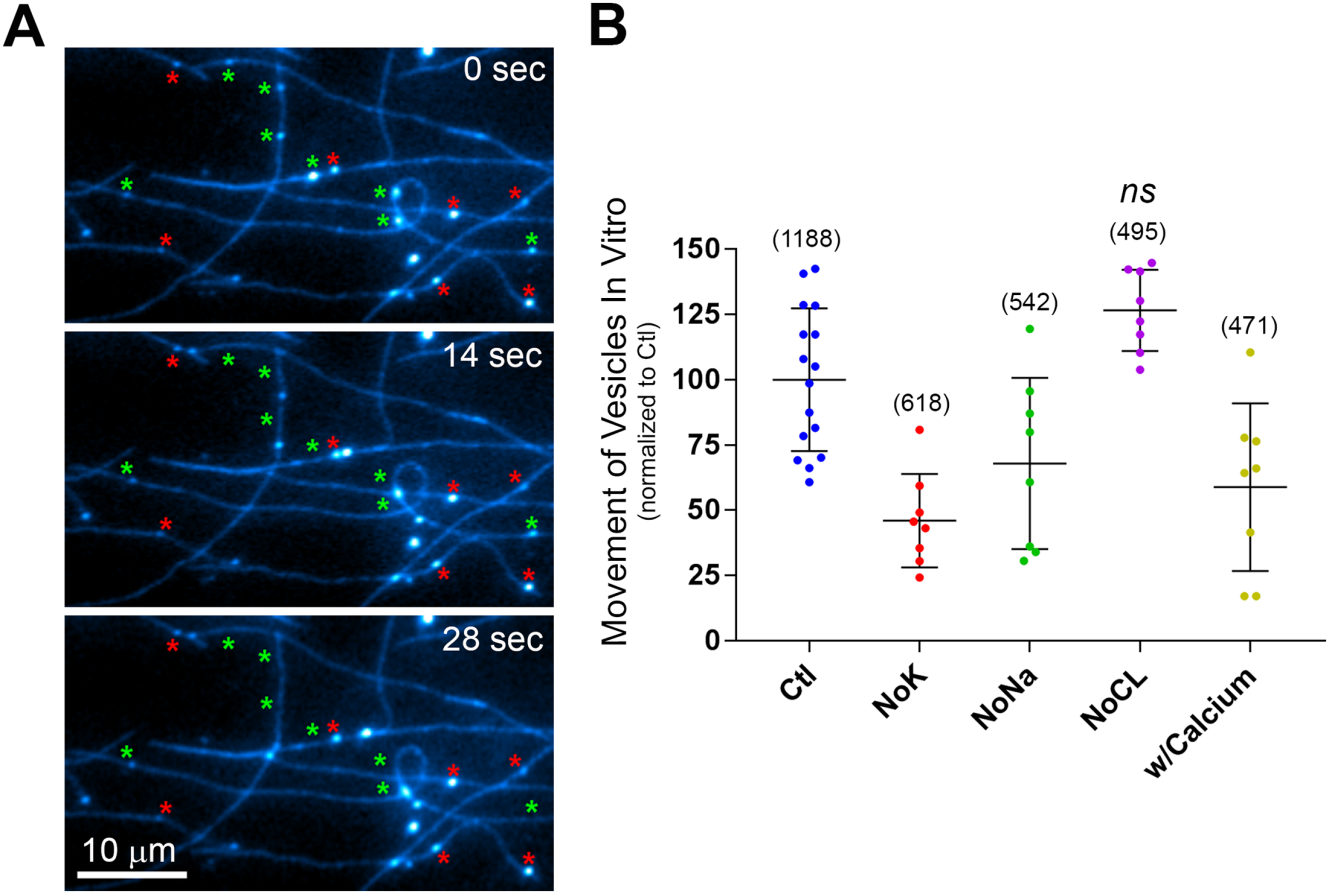
Cell free assays demonstrate that depletion of potassium or sodium or addition of calcium all inhibit the microtubule-based motility of endosomes. Rat livers were injected with fluorescent ASOR, a hepatocyte specific ligand, and fluorescent early endosomes were isolated and stored as frozen aliquots. These were thawed and added to purified microtubules within microscope imaging chambers. (**A)** A cropped region of a time lapse movie shows the appearance of endosomes (bright blue dots) bound to microtubules (blue filaments) during addition of ATP (0–28 sec). Green asterisks indicate vesicles that moved following ATP addition while red asterisks indicate vesicles that were stationary. (**B**) Quantification of vesicle movement under solute substitution, control (Ctl), elimination of potassium (NoK), sodium (NoNa), or chloride (NoCL), or with addition of 2 mM CaCl_2_ (w/Calcium). Each dot indicates a single experiment, bars are +/- SD, parentheses indicate total number of vesicles counted. Movement was normalized to control (100%) for clearer presentation. Average control movement was 48.2%. Means of all experiments were significantly different from control (p = 0.026 or less) except (*ns*), not significant, according to one way ANOVA followed by Dunnetts test.

Studies to this point had not provided a mechanism whereby media lacking particular solutes could induce a reduction in organelle motility. Under K^+^ depletion, Huh-7 cells were metabolically active, did not show obvious damage by propidium iodide and bright field imaging, and organelles looked grossly normal and stained strongly with dyes that depend on membrane polarity. To gain additional insight, we took advantage of in vitro motility assays that have been developed in our laboratory [32, 33]. In these assays, biochemically isolated fluorescent labeled organelles are allowed to bind purified fluorescent microtubules within microscope imaging chambers. Upon ATP addition, the organelles move along the microtubules using the motor proteins that are endogenously bound to the organelles. The amount of movement can be quantified as the percent of microtubule-bound vesicles that undergo ATP-dependent translocation during the 90 sec movies. Movement has been shown to be specific for the type of organelle, its protein constituents, and the presence of microtubule motor proteins [34, 35]. Magnesium-ATP is well known to be required for activity of the microtubule motors, dyneins and kinesins, but specific effects of K^+^ or Na^+^ are not well recognized beyond their effects on ionic strength. Several studies have indicated that motor proteins have increased motor activity in lower concentrations of KCl [36–38]. We predicted that substitution of K^+^ or Na^+^ or other solutes would have minimal effect, provided that ionic strength was maintained. Fluorescently labeled early endocytic vesicles were isolated from rat liver by injecting fluorescent ligand into the hepatic portal vein, homogenizing the liver and separating the organelles on Nycodenz density gradients according to a modified version of a procedure developed previously [33]. The fluorescent endocytic organelles were bound to microtubules in blocking buffer (35 mM Pipes-K_2_, 5 mM MgCl_2_, 1 mM EGTA, 0.5 mM EDTA, 4 mM DTT, 20 μM Taxol (paclitaxel), 2 mg/mL BSA, 5 mg/mL casein, pH 7.4) for 15 min and then washed in assay buffer (70 mM NaCl, 5 mM MgCl2, 5 mM KCl, 20 mM Hepes-Tris (12.5 mM Hepes, 7.5 mM Tris), 4 mM DTT, 10 μM Ouabain, 20 μM Taxol, pH 7.4) or assay buffer with solutes eliminated following the principles outlined in the previous sections (i.e. NaCl for KCl, choline-Cl^-^ for NaCl, gluconate salts and MgSO_4_ to eliminate Cl^-^). Motility was initiated by addition of 100 uM ATP in the same specific buffer and under time lapse fluorescent imaging. Fig 6A provides a representative region of imaging data from these experiments cropped to 213x120 pixels from the original 696x520. Typically 50–100 vesicles can be seen attached to microtubules in an entire field, and 40–50% of the attached vesicles move with addition of ATP (green asterisks). Eight to 16 experiments were performed for individual conditions, and these were scored by semi-automated analysis using macros written for ImageJ. Motility was scored as the percentage of microtubule-bound vesicles that moved ≥ 4 pixels (~one vesicle diameter). Motility did not occur in the absence of ATP. For control experiments, on average 48.2% +/- 13.8 (mean +/- SD, n = 16 experiments) of the vesicles moved; the spread of the data is apparent in the scatter plots of Fig 6B as each point represents a single experiment. The total number of vesicles counted is in parentheses. For clarity, the average motility for control experiments was set to 100%. As can be seen in Fig 6B, solute substitutions strongly affected in vitro motility. K^+^ depletion reduced the number of microtubule bound vesicles moving by 54% (p = 0.0001, one way ANOVA, Dunnets test), whereas Na^+^ depletion reduced motility by 32% (p = 0.026), and addition of Ca^2+^, which is typically absent in motility assays, reduced motility by 41% (p = 0.003). Extended incubation of microtubules in the presence of Ca^2+^ led to their depolymerization despite the presence of Taxol [39]. Calcium-containing experiments were therefore performed 3 min after Ca^2+^ addition, when microtubules were not noticeably altered. Interestingly, depletion of Cl^-^ increased motility by 26.5%, and this effect was consistent, although it did not achieve significance under Dunnett's multiple comparison test of the five experimental groups (p = 0.0805). Overall to our surprise, the solute composition of the cytosolic medium was critical for obtaining optimal translocation of the organelles along microtubules. We did not detect differences in the appearance of the endocytic organelles under different conditions. For instance, a similar average number of vesicles per field were bound to the microtubules (74.3, 77.3, 67.8, 61.9, 58.9 for Ctl, NoK, NoNa, NoCL, w/Calcium). Experiments used the same preparation of organelles and alternated between control and experimental groups during the experimental procedure. Thus it appears that the motile machinery itself, presumably motor proteins, coat proteins, or other molecules that interact with the motors, directly require these solutes for optimal activity. Removal of Na^+^ or K^+^, for example, does not release the vesicles from the microtubules, but rather results in their becoming more anchored, perhaps because of less active motors. In total the studies indicate that removal of K^+^ or Na^+^ from the external medium of living cells (Fig 3) or from the cytosolic medium of a purified component system (Fig 6) strongly reduces organelle traffic. The lowered motility within the in vitro system suggests that that a direct requirement of the solutes by the motile machinery may partially explain their requirement within the extracellular medium. It will be important to distinguish the molecular identities and specific nature of this requirement as well as the potential physiologic importance of lowered organelle motility in future studies.

## Discussion

This report investigates whether removal of specific solutes from extracellular medium can affect the trafficking of organelles. Previous studies had found that K^+^ depletion can inhibit endocytosis and the formation of cell polarity [17, 18, 40], but these did not examine organelle motility. Here we show that a relatively brief exposure of cells to K^+^ free medium strongly inhibits organelle trafficking. Under these conditions, cells were viable, showed unaltered microtubule cytoskeleton, and accumulated Lysotracker, TMRE, and DIBAC4(3) dyes, which depend on organelle and plasma membrane potential. We also found that extracellular medium lacking Na^+^ or Cl^-^ could reduce trafficking of FL-EGF containing organelles, and previous studies from our laboratory had shown that Na^+^ depletion can reduce the delivery of ligand to lysosomes [41]. Here we found that, under K^+^ depletion, mitochondrial oxygen consumption was similar but extracellular acidification was lowered, for instance by 22% under glucose stimulated conditions (47.91 +/- 10.3 vs 37.45 +/- 7.6 pmol/min, mean +/- SD, Ctl vs NoK, p<0.0001, n = 32, 4 time points, 8 wells). The latter observation suggests either reduced glycolysis or altered exchange of cellular protons. Experiments using isolated endosomes and purified microtubules showed that removal of K^+^ or Na^+^ also reduced motility in vitro. One simple explanation for these results is that K^+^ and Na^+^ ions are required for one or more components of the organelle motile machinery. Depletion in the medium would result in depletion within the cytosol and inhibition of motor proteins or alteration of organelle protein coats that are required for motor protein function. Interestingly and in contrast, depletion of Cl^-^ in the extra cellular medium caused inhibition of motility yet appeared to stimulate motility in the cell free assays (although at p = 0.0805, this did not reach significance by Dunnett's multiple comparison test). Chloride has been shown by several groups to partially inhibit dynein and kinesin microtubule motors in vitro [42–44], but as far as we are aware, K^+^ and Na^+^ are not specifically required by microtubule motors, and ciliary dynein appears to function in their absence [36]. K^+^ depletion, however, has been shown to inhibit the formation of proper clathrin lattices, potentially by inhibiting adapter-clathrin interactions [40, 45].

Several treatments have now been shown to inhibit both endocytosis and organelle motility. These include cytosolic acidification [46], hypertonic shock [47], and K^+^ depletion (Fig 1). Hypotonic shock, such as used in Fig 2, has not been shown to inhibit organelle motility. Although one might propose that toxicity from these treatments would cause mitochondrial dysfunction and ATP depletion or cellular damage, Figs 4 and 5 demonstrate normal mitochondria appearance, strong uptake of the membrane potential-dependent dye, TMRE, and a normal oxygen consumption in the mitochondrial stress test. In fact, the results agree with earlier research demonstrating that ATP levels are not strongly affected by short term K^+^ depletion [19, 31]; a survey of DNA and RNA synthesis, amino acid uptake, and cellular ATP levels, showed that protein synthesis was primarily affected [17, 19]. Intracellular acidification, unlike the other treatments, has been shown to redistribute lysosomes to the cell periphery, and it is possible that acidification preferentially inhibits the inward movement of organelles [45, 48]. Additionally, the internal pH of lysosomes at the cell periphery have been shown to be less acidic [49], and at present it is unclear how luminal pH may be coordinated with lysosome motility and the pH of the cytosol.

Dejonghe et al [46] suggest that proton ionophores, including mitochondrial uncouplers, can inhibit organelle motility and endocytosis by inducing cytosolic acidification through increased H^+^ import from the medium or from lysosomes. Previously our laboratory demonstrated that Na^+^ free medium induces cytosolic acidification, presumably by inhibiting extrusion of protons by the Na^+^/ H^+^ antiporter, and it was suggested that cytosolic acidification is responsible for reduced delivery of ligand to lysosomes [50]. Additionally, Cl^-^ antiporters and channels are regulators of intracellular pH, and we observed reduced FL-EGF organelle motility in Cl^-^ free medium (Fig 3). It therefore appears that reduced cytosolic pH can correlate with reduced organelle motility. But at present it is not known how the inhibition of motility is achieved. Perhaps in conflict with the above observations, one report showed only moderate (~0.1 unit) cytosol acidification from K^+^ depletion [51], and hypertonic medium, which also inhibits organelle motility, has been shown to stimulate the Na^+^/ H^+^ antiporter and alkalinize the cytosol [52]. Interestingly, we saw slightly reduced *extracellular* acidification under K^+^ depletion using the Seahorse XF analyzer (Fig 5). This could occur if the Na^+^/ H^+^ antiporter was inhibited or if cytosol was alkaline.

There appears to be considerable variation in the effects of K^+^ depletion between cell types. Previous studies have shown large differences in the rate of K^+^ depletion and its physiologic effects for different cell lines following its removal from medium [17, 19], and these differences may reflect important underlying phenotypes. Table 1 lists markedly different changes in DIBAC4(3) staining, a measure of plasma membrane polarity, under the same K^+^ free treatment. Removal of K^+^ should have the simultaneous effect of promoting hyperpolarization through outward leak of K^+^, the primary cytosolic cation, and promoting depolarization by inhibiting the Na^+^/ K^+^ ATPase. Since motility was inhibited for all cells, a specific change in plasma membrane polarity does not appear to be critical. Mitochondrial staining appeared more consistent (Table 1), in that all cell lines showed some degree of increased TMRE under K^+^ depletion, indicating hyperpolarization. This might be explained by decreased mitochondrial K^+^(ATP) channel current, which normally serves to reduce or normalize mitochondrial hyperpolarization [53]. It is unclear whether mitochondrial hyperpolarization is involved in reduced organelle motility. As mentioned, Dejonghe et al [46] saw reduced organelle motility in the presence of proton ionophores, which should be depolarizing. We did not detect major differences in the oxygen consumption rate (OCR) of Huh-7 cells under K^+^ depletion (Fig 5), suggesting that if mitochondria are hyperpolarized that ATP production is held in check, as would occur under limiting levels of ADP. Interestingly, reduced organelle motility should lead to reduce hydrolysis of cellular ATP and potentially lower levels of ADP. We did detect a 10.0% increase in OCR following treatment with the uncoupler, FCCP, under K^+^ depletion (84.70 +/- 18.4 vs 93.14 +/- 18.1 pmol/min, Ctl vs NoK, mean +/- SD, p = 0.03, n = 44, 4 time points, 11 wells), which is consistent with hyperpolarized mitochondria that was indicated by the 14.1% increase in TMRE fluorescence (Table 1). On close inspection of Table 1 it is also interesting to note that the changes in positively charged TMRE and negatively charged DIBAC4(3) intensity are inversely correlated as would be expected for dyes that depend on charge for their accumulation (i.e. that follow Nernstian behavior). For instance, HeLa cells showed a less electronegative change in the cytosol compared to the other cells under K^+^ depletion (i.e. showed a weaker % increase in TMRE and greater % increase in DIBAC4(3)).

Mitochondrial motility was decreased under low K^+^ (Fig 1, Table 1), but oxidative phosphorylation for Huh-7 cells appeared relatively unaffected (Fig 5). Experimental treatments that reduce mitochondrial fission and fusion, depolymerize microtubules, or those that inhibit mitochondria energetics can alter mitochondria morphology and distribution over time. Such treatments are often associated with decreased oxidative phosphorylation as well as the mislocalization of mitochondria, especially in neuronal cells [3, 54, 55]. However, short term reduction in the motility of mitochondria is not necessarily associated with decreased respiration. For instance, protein anchoring and calcium induced pausing of mitochondria appear to be necessary for accumulation of actively respiring mitochondria in growth cones and synapses [54, 55]. Slightly higher motility has been observed in mitochondria with lower membrane potential and more oxidized redox potential [56], and addition of the respiration inhibitor, antimycin, can actually increase mitochondria motility [57]. Here we show that K^+^ free medium reduces mitochondrial movement but does not strongly affect oxidative phosphorylation for Huh-7 cells in the 90+ minutes of observation. Although we have not yet measured any effects on fission and fusion, we expect that mitochondria function will eventually decrease, since we observed cell death within 18 hours (Fig 3). We also expect that the rate and extent of mitochondria changes would depend on the cell type (Table 1).

With regard to ATP levels, we show that oxidative phosphorylation and glycolysis do not appear strongly affected by K^+^ depletion in Huh7 cells and that they remain responsive to metabolic regulation (Fig 5). Furthermore, previous studies have found that ATP levels are not strongly affected by the depletion of K^+^ in cultured cells [19, 31]. We did observe a ~22% decrease in ECAR, the indicator of glycolysis, under K^+^ depletion (Fig 5) that could reflect reduced ATP. However, it is not clear whether reduced glycolysis affects organelle motility. In experiments of Fig 3 we eliminated glucose from the medium, which will also reduce glycolysis, and did not observe a reduction in motility. It is possible however, that moderate reductions in ATP could reduce organelle motility when K^+^ is also depleted. Surprisingly, it is not well documented whether relatively small changes to cellular ATP levels can affect organelle motility. The affinities of motor proteins for ATP are generally in the 2–60 micromolar range [58, 59], whereas cells typically contain approximately 5 mM ATP [60], and 100 uM ATP provides optimal motility of organelles for in vitro assays (Fig 6; [1]). This suggests that > 50 fold reduction in cellular ATP may be required to affect organelle motility. Nonetheless, different requirements may exist within intact cytosol.

For these studies, both living cell and in vitro motility assays demonstrated a significant reduction in organelle motility in the absence of K^+^ and Na^+^, and this suggests that any changes in membrane polarities or chemical gradients within the cell may not be vital to the observed reduction in motility. Rather it is possible that motor proteins or associated molecules specifically require K^+^ and Na^+^ for optimal activity and that these molecules could be regulated by changes in intracellular concentrations of these solutes. This concept requires significant future investigation as it is unclear whether solute concentrations could change sufficiently to alter organelle traffic physiologically. Potentially relevant scenarios include ischemic injury, apoptotic or necrotic stimuli, as well as exposure to drugs (e.g. ouabain) that are known to induce intracellular K^+^ depletion and changes in other solutes. The level of endocytosis and organelle trafficking may play an important role in recovery from such stress, and this could be especially relevant for neurons, which rely heavily on intracellular trafficking. For instance, inhibition of mitochondrial motility could contribute to cell death by inhibiting fission and fusion and the associated ability to self repair [3]. The utilization of cell free assays has suggested that direct effects of the solutes on motile machinery should at least be considered, and identifying the specific targets that are affected and physiologic circumstances where such changes may take place could provide new insight into the control of organelle traffic.

## Supporting information

S1 Movie Movement of FL-EGF organelles in live cell (control) medium for Huh-7 cells. Cells were subject to the live cell organelle motility protocol with fluorescent EGF (white) and Hoechst stained nuclei (cyan), 1 min real time, 31 frames.
(TIF)

S2 Movie Movement of FL-EGF organelles in potassium free (NoK) live cell medium for Huh-7 cells. Cells were subject to the live cell organelle motility protocol with fluorescent EGF (white) and Hoechst stained nuclei (cyan), 1 min real time, 31 frames.
(TIF)

S3 Movie Movement of GFP-LC3 organelles in live cell (control) medium for 3T3 cells. Cells that had been transfected with mCherry-GFP-LC3 were subject to the live cell organelle motility protocol without addition of FL-EGF. GFP-LC3 fluorescence channel is shown (white) with Hoechst stained nuclei (cyan), 1 min real time, 31 frames.
(TIF)

S4 Movie Movement of GFP-LC3 organelles in potassium free (NoK) live cell medium for 3T3 cells. Cells that had been transfected with mCherry-GFP-LC3 were subject to the live cell organelle motility protocol without addition of FL-EGF. GFP-LC3 fluorescence channel is shown (white) with Hoechst stained nuclei (cyan), 1 min real time, 31 frames.
(TIF)

S5 Movie Movement of TMRE labeled mitochondria in live cell (control) medium for Huh-7 cells. Cells were subject to the live cell organelle motility protocol with addition of 30 nM TMRE (white) prior to imaging, as described in materials and methods. The brightness was enhanced (normalized) to highlight dimmer staining organelles making some of the fluorescence appear saturated (bright white). Original images are not saturated, 1 min real time, 31 frames.
(TIF)

S6 Movie Movement of TMRE labeled mitochondria in potassium free (NoK) live cell medium for Huh-7 cells. Cells were subject to the live cell organelle motility protocol with addition of 30 nM TMRE (white) prior to imaging, as described in materials and methods. The brightness was enhanced (normalized) to highlight dimmer staining organelles making some of the fluorescence appear saturated (bright white). Original images are not saturated, 1 min real time, 31 frames.
(TIF)

S1 Fig Appearance of different cell lines exposed to control and potassium free medium. Cells were exposed to FL-EGF (EGF) or Lysotracker (LysoTr) or stably transfected with mCherry-GFP-LC3 (LC3) and exposed to Hoechst nuclear stain and then 90 min of live cell medium (Ctl, left panels) or K^+^ free medium (NoK, right pannels) and then imaged. Representative bright field (gray) or fluorescence (dark) images of different fields of cells demonstrate the appearance of cells and the putative lysosome array (or autophagosomes for LC3, GFP channel) in 5 cell lines. Fluorescence images were normalized to highlight dimmer staining organelles making the images appear saturated (white). The original images are not saturated. LC3 GFP images reveal significant cytosolic, diffuse staining, which is presumably due to the soluble form of this protein. 3T3 and MDCK cells showed contraction of the cell membrane with exposure to K^+^ free medium.
(TIF)

S2 Fig Appearance of Huh-7 cells treated with media lacking potassium, sodium, chloride, magnesium, calcium, or glucose or medium lacking potassium and the other solutes. Huh-7 cells were exposed to FL-EGF (EGF) followed by Hoechst nuclear stain and then 90 min of live cell medium (Ctl) or medium lacking the solutes indicated. Solutes were substituted as described in materials and methods. Chloride free as well as Ca^+2^ free medium resulted in contraction of the cytoplasm and a more focused, centrally located FL-EGF array.
(TIF)

S3 Fig Reduced movement of FL-EGF organelles in potassium free Seahorse assay medium. Cells were subject to the live cell organelle motility protocol using live cell medium, +/- K^+^ (Ctl and NoK) and mitochondria stress tests assay buffer, +/- K^+^ (Seahorse and NoK Seahorse), which contains NaH_2_PO_4_, glutamine, Na pyruvate but no other buffering reagents. Motility was decreased when K^+^ was removed from either medium. Each dot represents a field of cells with 3 experiments for each condition. Bars are mean +/- SD.
(TIF)
