# Supplementary figures and images for: Reduction of organelle motility by removal of potassium and other solutes
Source: PLoS One. 2017 Sep 18;12(9):e0184898. doi: 10.1371/journal.pone.0184898 (PMC5602639; doi:10.1371/journal.pone.0184898)

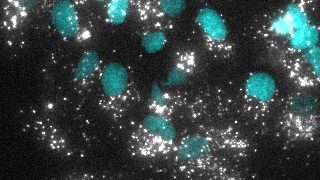

Supplement: S1 Movie — Cells were subject to the live cell organelle motility protocol with fluorescent EGF (white) and Hoechst stained nuclei (cyan), 1 min real time, 31 frames. (TIF) [file pone.0184898.s001.tif]

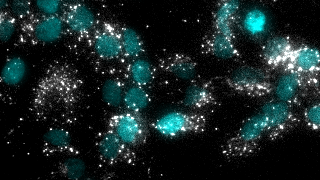

Supplement: S2 Movie — Cells were subject to the live cell organelle motility protocol with fluorescent EGF (white) and Hoechst stained nuclei (cyan), 1 min real time, 31 frames. (TIF) [file pone.0184898.s002.tif]

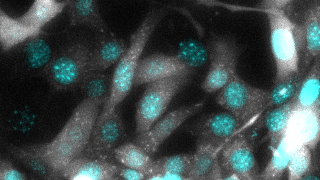

Supplement: S3 Movie — Cells that had been transfected with mCherry-GFP-LC3 were subject to the live cell organelle motility protocol without addition of FL-EGF. GFP-LC3 fluorescence channel is shown (white) with Hoechst stained nuclei (cyan), 1 min real time, 31 frames. (TIF) [file pone.0184898.s003.tif]

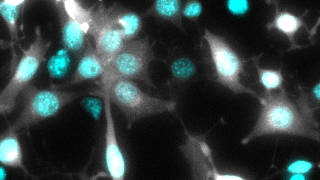

Supplement: S4 Movie — Cells that had been transfected with mCherry-GFP-LC3 were subject to the live cell organelle motility protocol without addition of FL-EGF. GFP-LC3 fluorescence channel is shown (white) with Hoechst stained nuclei (cyan), 1 min real time, 31 frames. (TIF) [file pone.0184898.s004.tif]

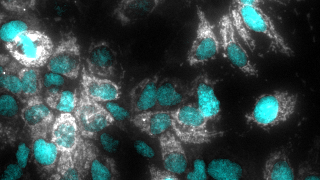

Supplement: S5 Movie — Cells were subject to the live cell organelle motility protocol with addition of 30 nM TMRE (white) prior to imaging, as described in materials and methods. The brightness was enhanced (normalized) to highlight dimmer staining organelles making some of the fluorescence appear saturated (bright white). Original images are not saturated, 1 min real time, 31 frames. (TIF) [file pone.0184898.s005.tif]

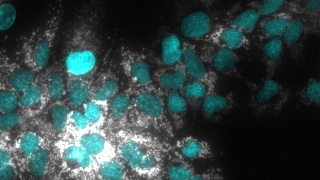

Supplement: S6 Movie — Cells were subject to the live cell organelle motility protocol with addition of 30 nM TMRE (white) prior to imaging, as described in materials and methods. The brightness was enhanced (normalized) to highlight dimmer staining organelles making some of the fluorescence appear saturated (bright white). Original images are not saturated, 1 min real time, 31 frames. (TIF) [file pone.0184898.s006.tif]

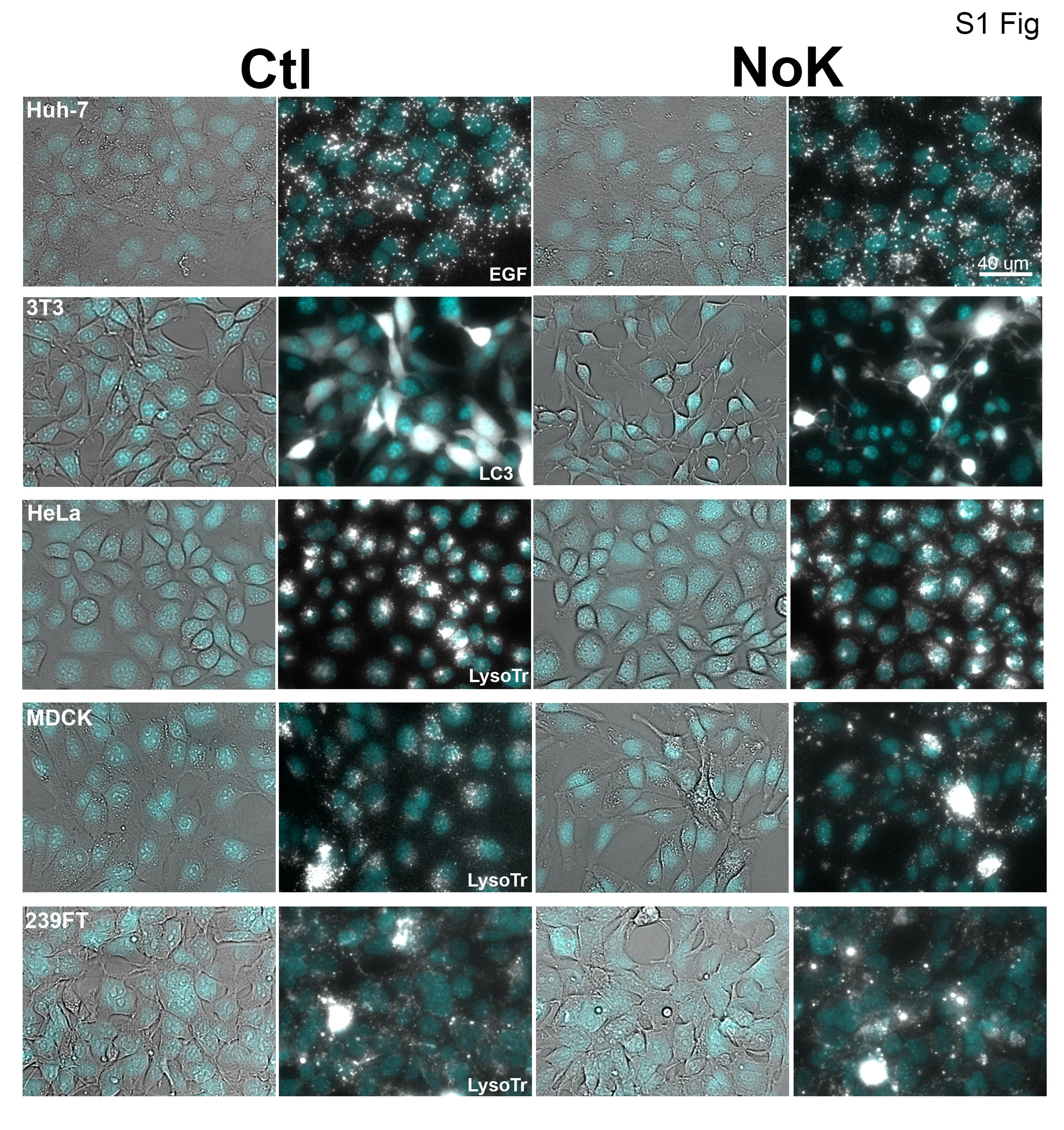

Supplement: S1 Fig — Cells were exposed to FL-EGF (EGF) or Lysotracker (LysoTr) or stably transfected with mCherry-GFP-LC3 (LC3) and exposed to Hoechst nuclear stain and then 90 min of live cell medium (Ctl, left panels) or K+ free medium (NoK, right pannels) and then imaged. Representative bright field (gray) or fluorescence (dark) images of different fields of cells demonstrate the appearance of cells and the putative lysosome array (or autophagosomes for LC3, GFP channel) in 5 cell lines. Fluorescence images were normalized to highlight dimmer staining organelles making the images appear saturated (white). The original images are not saturated. LC3 GFP images reveal significant cytosolic, diffuse staining, which is presumably due to the soluble form of this protein. 3T3 and MDCK cells showed contraction of the cell membrane with exposure to K+ free medium. (TIF) [file pone.0184898.s007.tif]

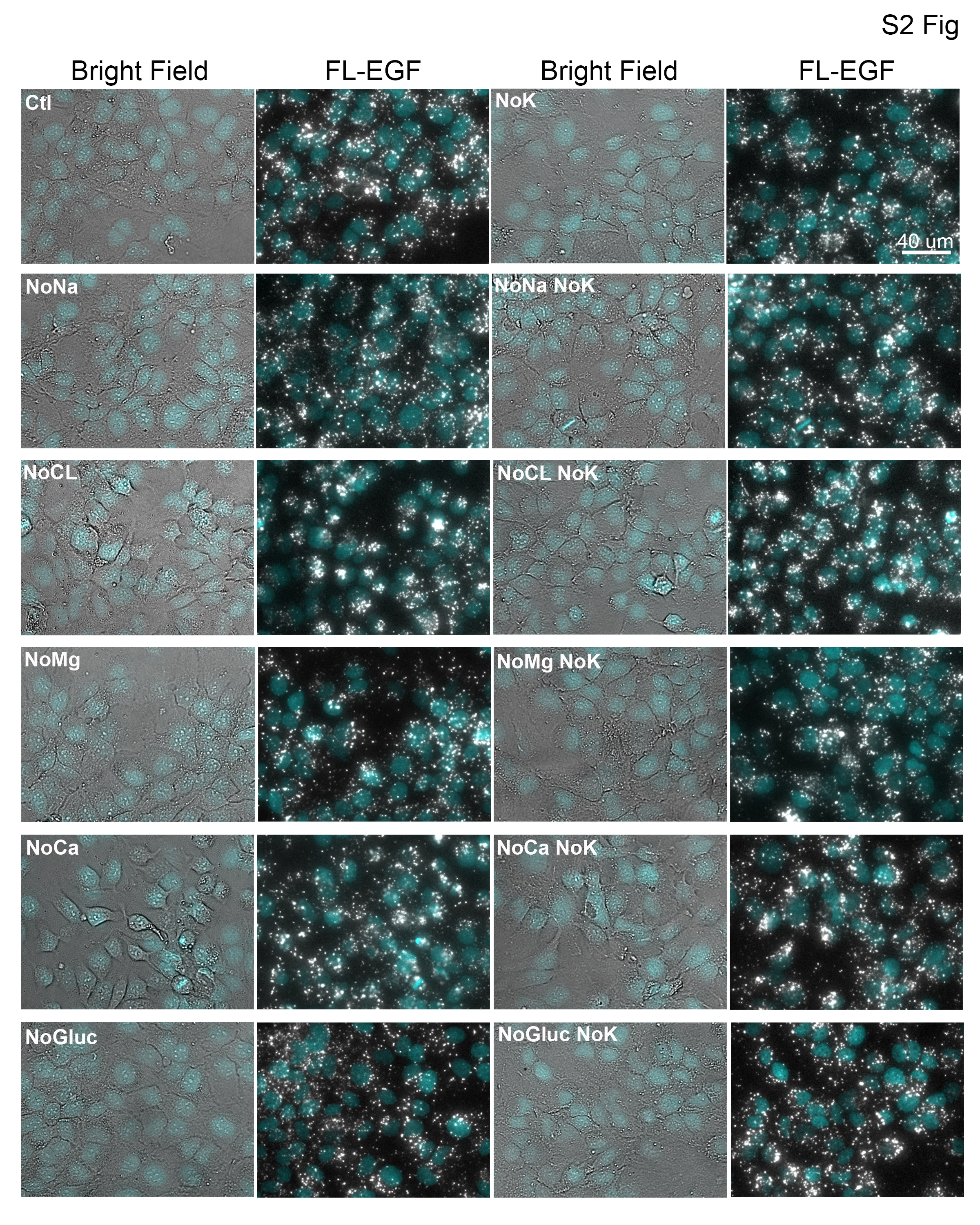

Supplement: S2 Fig — Huh-7 cells were exposed to FL-EGF (EGF) followed by Hoechst nuclear stain and then 90 min of live cell medium (Ctl) or medium lacking the solutes indicated. Solutes were substituted as described in materials and methods. Chloride free as well as Ca+2 free medium resulted in contraction of the cytoplasm and a more focused, centrally located FL-EGF array. (TIF) [file pone.0184898.s008.tif]

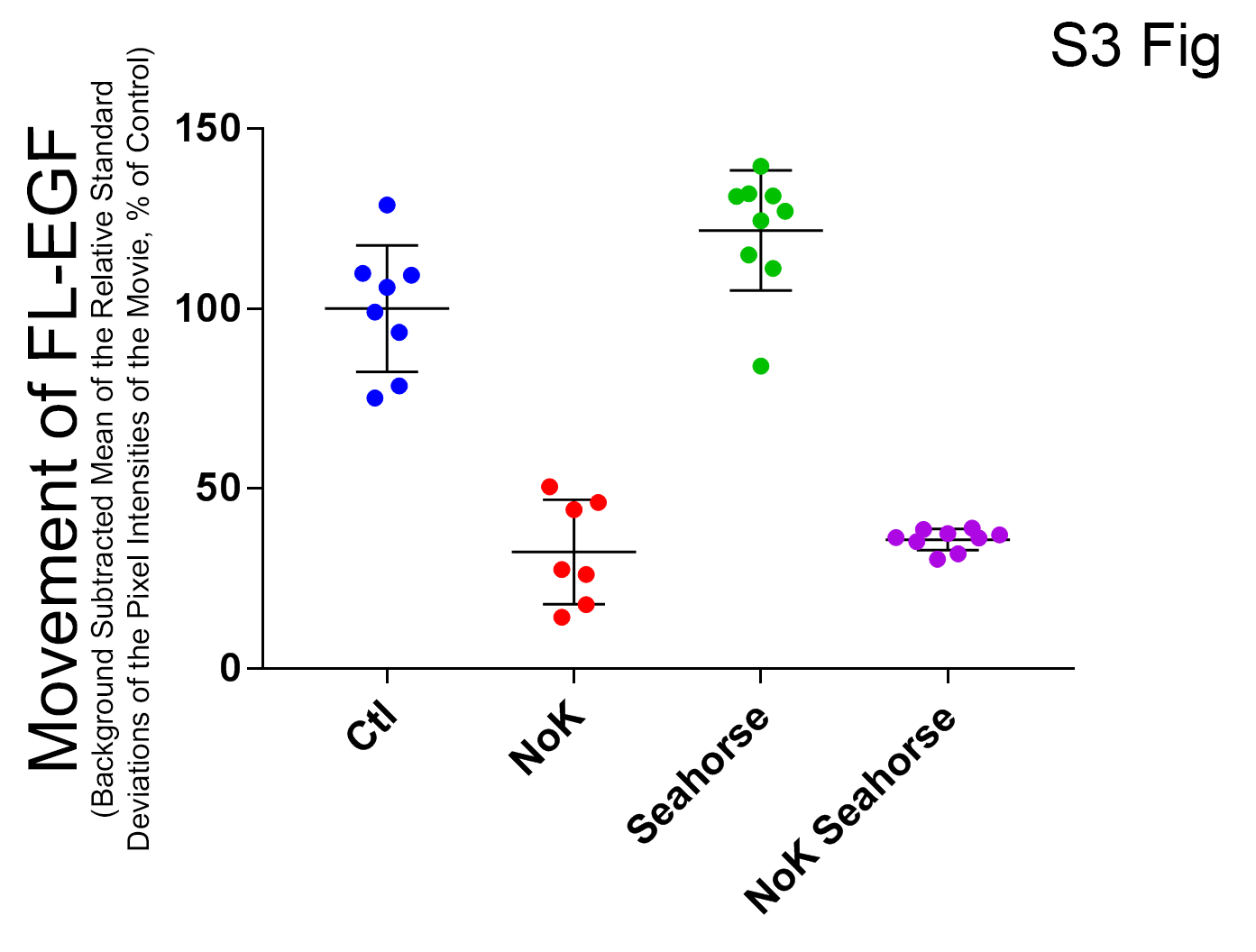

Supplement: S3 Fig — Cells were subject to the live cell organelle motility protocol using live cell medium, +/- K+ (Ctl and NoK) and mitochondria stress tests assay buffer, +/- K+ (Seahorse and NoK Seahorse), which contains NaH2PO4, glutamine, Na pyruvate but no other buffering reagents. Motility was decreased when K+ was removed from either medium. Each dot represents a field of cells with 3 experiments for each condition. Bars are mean +/- SD. (TIF) [file pone.0184898.s009.tif]
